# RNA sequencing unravels novel L cell constituents and mechanisms of GLP-1 secretion in human gastric bypass-operated intestine

**DOI:** 10.1007/s00125-023-06046-8

**Published:** 2023-11-30

**Authors:** Michael G. Miskelly, Andreas Lindqvist, Elena Piccinin, Alexander Hamilton, Elaine Cowan, Bent-Johnny Nergård, Rita Del Giudice, Mtakai Ngara, Luis R. Cataldo, Dmytro Kryvokhyzha, Petr Volkov, Luke Engelking, Isabella Artner, Jens O. Lagerstedt, Lena Eliasson, Emma Ahlqvist, Antonio Moschetta, Jan Hedenbro, Nils Wierup

**Affiliations:** 1 Neuroendocrine Cell Biology, Lund University Diabetes Centre, Lund University, Malmö, Sweden; 2 Department of Translational Biomedicine and Neuroscience, University of Bari ‘Aldo Moro’, Bari, Italy; 3 Department of Interdisciplinary Medicine, University of Bari ‘Aldo Moro’, Bari, Italy; 4 Molecular Metabolism, Lund University Diabetes Centre, Lund University, Malmö, Sweden; 5 Islet Cell Exocytosis, Lund University Diabetes Centre, Lund University, Malmö, Sweden; 6 Aleris Obesitas, Lund, Sweden; 7 Department of Experimental Medical Science, Lund University, Lund, Sweden; 8 Department of Biomedical Science and Biofilms – Research Center for Biointerfaces, Malmö University, Malmö, Sweden; 9 Novo Nordisk Foundation Centre for Basic Metabolic Research, Faculty of Health and Medical Sciences, University of Copenhagen, Copenhagen, Denmark; 10 Bioinformatics Unit, Lund University Diabetes Centre, Lund University, Malmö, Sweden; 11 Internal Medicine, University of Texas Southwestern Medical Center, Dallas, TX USA; 12 Department of Molecular Genetics, University of Texas Southwestern Medical Center, Dallas, TX USA; 13 Endocrine Cell Differentiation and Function, Stem Cell Centre, Lund University, Malmö, Sweden; 14 Genomics, Diabetes and Endocrinology, Lund University Diabetes Centre, Lund University, Malmö, Sweden; 15 INBB National Institute for Biostructure and Biosystems, Rome, Italy; 16 Department of Surgery, Department of Clinical Sciences Lund, Lund University, Lund, Sweden

**Keywords:** Gastric bypass surgery, GLP-1, Glucagon-like peptide-1, Intestine, Obesity, Remission, RNA sequencing, SCD, Stearoyl-CoA desaturase, Type 2 diabetes

## Abstract

**Aims/hypothesis:**

Roux-en-Y gastric bypass surgery (RYGB) frequently results in remission of type 2 diabetes as well as exaggerated secretion of glucagon-like peptide-1 (GLP-1). Here, we assessed RYGB-induced transcriptomic alterations in the small intestine and investigated how they were related to the regulation of GLP-1 production and secretion in vitro and in vivo.

**Methods:**

Human jejunal samples taken perisurgically and 1 year post RYGB (*n*=13) were analysed by RNA-seq. Guided by bioinformatics analysis we targeted four genes involved in cholesterol biosynthesis, which we confirmed to be expressed in human L cells, for potential involvement in GLP-1 regulation using siRNAs in GLUTag and STC-1 cells. Gene expression analyses, GLP-1 secretion measurements, intracellular calcium imaging and RNA-seq were performed in vitro. OGTTs were performed in C57BL/6j and i*Scd1*^−/−^ mice and immunohistochemistry and gene expression analyses were performed ex vivo.

**Results:**

Gene Ontology (GO) analysis identified cholesterol biosynthesis as being most affected by RYGB. Silencing or chemical inhibition of stearoyl-CoA desaturase 1 (SCD1), a key enzyme in the synthesis of monounsaturated fatty acids, was found to reduce *Gcg* expression and secretion of GLP-1 by GLUTag and STC-1 cells. *Scd1* knockdown also reduced intracellular Ca^2+^ signalling and membrane depolarisation. Furthermore, *Scd1* mRNA expression was found to be regulated by NEFAs but not glucose. RNA-seq of SCD1 inhibitor-treated GLUTag cells identified altered expression of genes implicated in ATP generation and glycolysis. Finally, gene expression and immunohistochemical analysis of the jejunum of the intestine-specific *Scd1* knockout mouse model, i*Scd1*^−/−^, revealed a twofold higher L cell density and a twofold increase in *Gcg* mRNA expression.

**Conclusions/interpretation:**

RYGB caused robust alterations in the jejunal transcriptome, with genes involved in cholesterol biosynthesis being most affected. Our data highlight SCD as an RYGB-regulated L cell constituent that regulates the production and secretion of GLP-1.

**Graphical Abstract:**

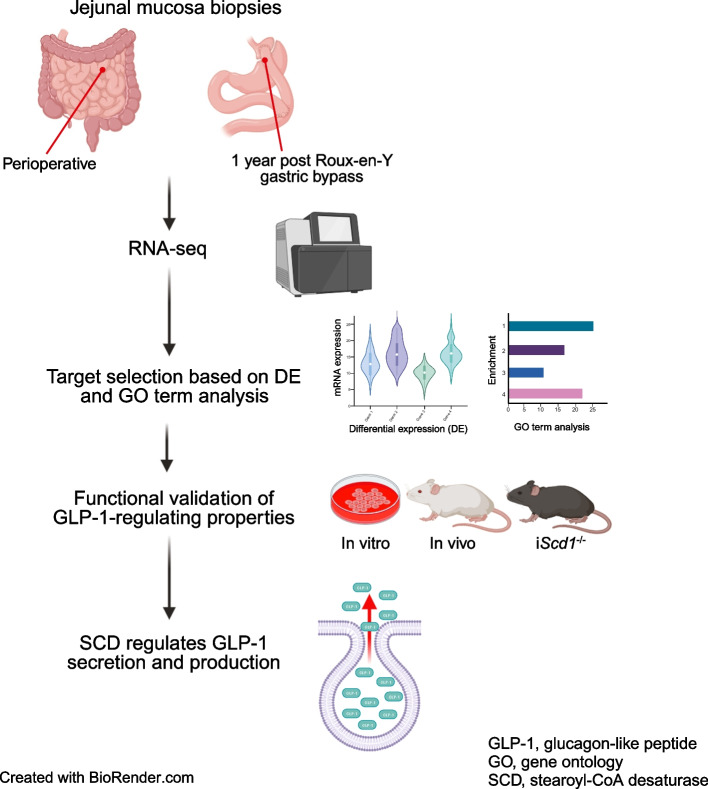

**Supplementary Information:**

The online version contains peer-reviewed but unedited supplementary material available at 10.1007/s00125-023-06046-8.


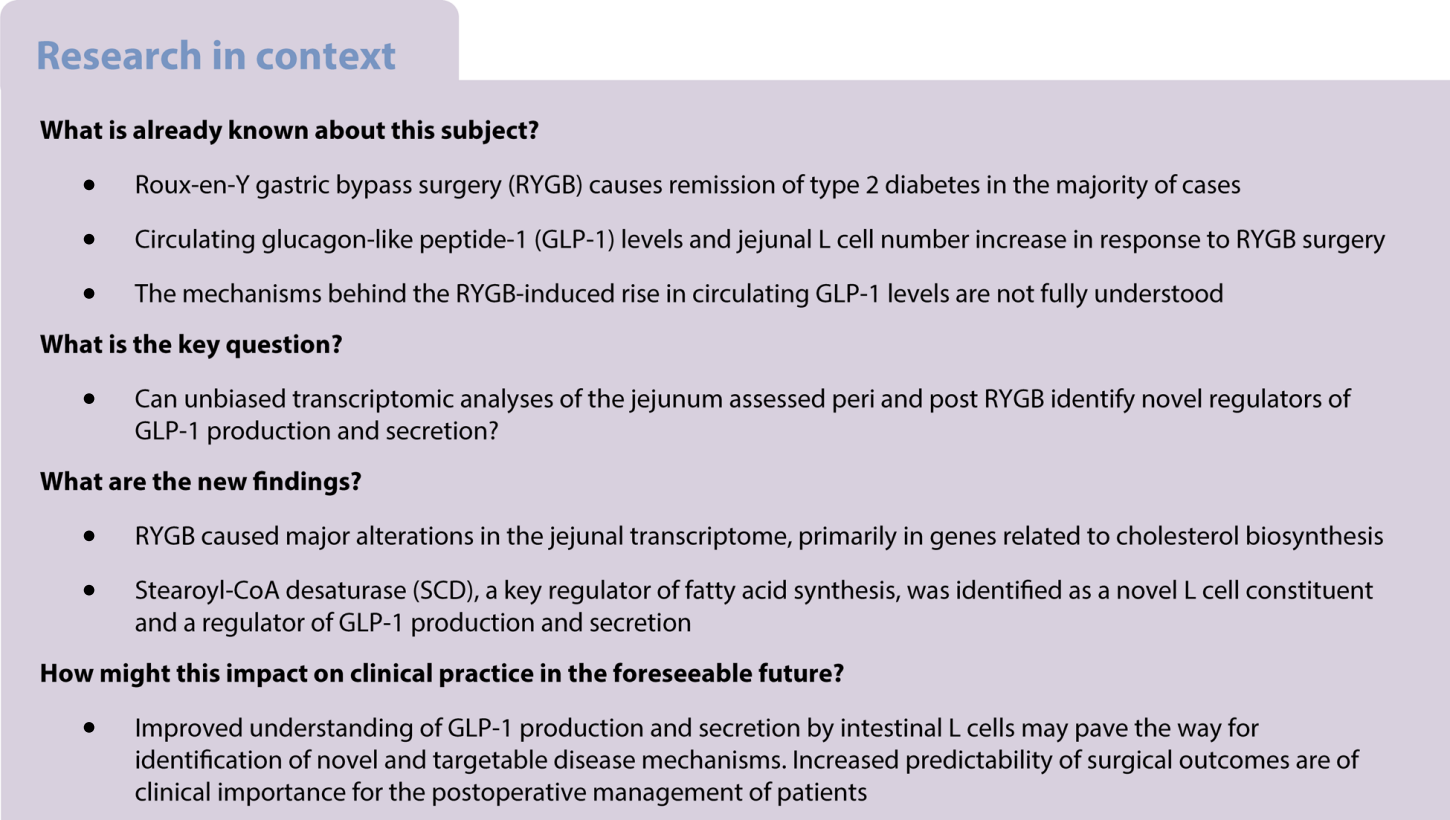


## Introduction

Roux-en-Y gastric bypass surgery (RYGB) results in remission of type 2 diabetes in the majority of cases [1, 2]. Several hypotheses have been proposed to explain the mechanism by which type 2 diabetes remission is achieved post surgery [3]. The hypothesis receiving the most attention focuses on amplified postprandial release of the incretin hormone glucagon-like peptide 1 (GLP-1) post RYGB [3]. Although increased postprandial GLP-1 levels in response to surgery is well established [4], and L cell density in the small intestine is robustly increased post RYGB [5], the underlying mechanisms remain poorly understood. We have shown that alterations in incretin secretion in response to surgery are likely to be independent of weight loss or food intake and, furthermore, to be unrelated to alterations in the metabolome [6, 7]. Bile acid alterations have been proposed to cause increased GLP-1 secretion [8] via Takeda G-protein receptor 5 (TGR5) signalling [9] and GLP-1 levels have been linked to IL-6 levels [10]. However, the transcriptomic influences on the RYGB-induced increase in GLP-1 secretion from the intestine has yet to be described. In order to address this, we performed RNA-seq of jejunal mucosal samples taken perioperatively and postoperatively from individuals with a BMI >50 kg/m^2^. The jejunum is a part of the intestine where representative specimens of the mucosa can be procured at the same level in all individuals and with precision, both peri- and postoperatively. We targeted RYGB-affected genes involved in cholesterol biosynthesis and assessed their effects on *Gcg* expression and GLP-1 secretion in in vitro and in vivo models. Alongside C57Bl/6j mice, we used an intestinal-specific knockout model, i*Scd1*^−/−^ [11].

## Methods

### Human specimens

Laparoscopic RYGB was performed as previously described [5]. Jejunal specimens were collected from 13 human individuals with obesity, perisurgically during laparoscopic RYGB and 1 year postsurgically using gastroscopy as described previously [5]. Study participants had a BMI >50 kg/m^2^ and were not diagnosed with type 2 diabetes at the time of surgery. Information on the study participants is provided in Table 1.

**Table 1:** Anthropometrics of the study participants

Characteristic	Mean (min.–max.)
Age (years)	36.5 (20–62)
Height (cm)	175.7 (159–197)
Preoperative weight (kg)	175.2 (127–225)
Postoperative weight (kg)	111.3 (83–139)
Weight loss (kg)	63.9 (35–114)
Preoperative BMI (kg/m^2^)	56.4 (49.4–64.1)
Postoperative BMI (kg/m^2^)	36.0 (28.3–41.9)
BMI change (kg/m^2^)	20.3 (12.3–29.4)
Preoperative HbA_1c_ (mmol/mol)	41.3 (29.0–75.0)
Preoperative HbA_1c_ (%)	5.9 (4.8–9.0)
Postoperative HbA_1c_ (mmol/mol)	29.5 (23.0–40.0)
Postoperative HbA_1c_ (%)	4.8 (4.3–5.8)
HbA_1c_ change (mmol/mol)	11.9 (3.0–35.0)
Sex (male/female), *n*	7/6
Biliopancreatic limb length (60 cm/200 cm), *n*	6/7

### Immunohistochemistry

Immunohistochemistry was performed as previously detailed [5] using the following primary antibodies: rabbit anti-CHGA (1:400, SP-1, ImmunoStar, Hudson, WI, USA), guinea pig anti-glucagon (1:2500, M8707, Euro-Diagnostica, Malmö, Sweden), goat anti-GIP (1:500, sc-23554, Santa Cruz Biotechnology, Dallas, TX, USA), rabbit anti-INSIG1 (1:100, ab70784, Abcam, Cambridge, UK) rabbit anti-SCD1 (1:250, #2794, Cell Signalling Technology, Danvers, MA, USA), rabbit anti-SQLE (1:100, Life Technologies, Waltham, MA, USA) and rabbit anti-SREBP2 (1:1000, ab28482, Abcam, Cambridge, UK). The secondary antibodies (dilution 1:400) used were as follows: Cy^TM^2 Affinipure donkey anti-rabbit IgG (711225152), Cy^TM^2 Affinipure donkey anti-guinea pig IgG (706225148), Cy^TM^5 Affinipure donkey anti-guinea pig IgG (706175148) and Cy^TM^2 Affinipure donkey anti-goat IgG (705225147; all Jackson ImmunoResearch Europe, Ely, UK). Primary and secondary antibodies were diluted in 0.25% BSA and 0.25% Triton X-100 in PBS. Antibodies were validated by staining alongside a negative control (0.25% BSA and 0.25% Triton X-100 without the antibody) as well as testing in positive control tissue (e. g. liver for SCD).

### RNA sequencing

Total RNA was extracted from jejunal specimens and GLUTag cells as per the manufacturer’s instructions (Nucleo Spin RNA II, Macherey Nagel, Bethlehem, PA, USA). Following extraction, RNA libraries were generated using the Illumina TruSeq Stranded Total RNA Prep with Ribo-Zero Plus (Illumina, San Diego, CA, USA) protocol following the manufacturer’s recommendations. Briefly, libraries were prepared and sequencing was performed using the Illumina NextSeq 500/550 High Output Kit v2.5 on an Illumina NextSeq 500 instrument (75 bp, paired end). The quality of sequences was assessed with FastQC 0.11.9 [12] and summarised with MultiQC 1.10.1 [13]. Sequences were mapped and counted with Salmon 1.5.0 [14]. Differential gene expression analysis was performed using DESeq2 1.30.1 [15].

### Pathway analysis

To assess the functionality of differentially expressed genes (*p*_adj_<0.05), a PANTHER (protein analysis through evolutionary relationships) over-representation analysis was carried out using PANTHER software (http://pantherdb.org/; version 14.1) [16–18]. The list of differentially expressed genes was assessed for biological process and ranked by hierarchical clustering. For RNA-seq of stearoyl-CoA desaturase 1 (SCD1) inhibitor-treated GLUTag cells, we performed Gene Ontology (GO) term analysis using PANTHER (version 16.0) on false discovery rate (FDR)-corrected significantly differentially expressed genes with at least 1.2-fold up- or downregulation.

### Cell culture

GLUTag cells were kindly provided by D. J. Drucker (Mount Sinai Hospital, Toronto, Canada) and used for gene expression and secretory assays. Cells were cultured as described previously [19]. STC-1 cells were a kind gift from J. Y. Scoazec, (Edouard Herriot Hospital, Lyon, France) and used for gene expression studies. Briefly, cells were cultured in DMEM containing 4.5 g/l glucose and supplemented with 10% FBS (vol./vol.) and antibiotics (100 U/ml penicillin and 0.1 mg/l streptomycin; Sigma Aldrich, St. Louis, MO, USA). Cells were tested for mycoplasma contamination using the Lonza MycoAlert Mycoplasma Detection Kit (LT07-418, Lonza, Basel, Switzerland). All cell-based assays were performed in duplicate and repeated in six different passages of cells.

### siRNA-mediated gene knockdown

GLUTag cells were seeded in 24-well plates with 250,000 cells per well. Gene knockdown (KD) was performed using Lipofectamine RNAiMAX (Life Technologies). siRNA (Silencer Select Pre-designed siRNA; Ambion, Life Technologies) targeting *Insig1* (s106741), *Scd1* (s73341), *Srebf2* (s74389) and *Sqle* (s74373) in the mouse genome were transfected at 50 pmol/l as per the manufacturer’s protocol. Scrambled siRNA (4390844) was used as a negative control (Silencer Select Negative Control No. 1 siRNA: Ambion, Life Technologies). RNA was extracted 48 h after KD as per the manufacturer’s instructions (Nucleo Spin RNA II, Macherey Nagel, Dueren, Germany). The effects of gene KD on apoptosis, cell viability and cytotoxicity were assessed using the ApoTox-Glo Triplex Assay (Promega, Madison, WI, USA) as per the manufacturer’s instructions.

### Incubation of GLUTag cells with fatty acids and glucose

Cells were seeded as described above and incubated at 37°C and 5% CO_2_ for 24 h. Following this, the medium was replaced with normal growth medium or DMEM with 10% FBS (vol./vol.) including glucose (2.8 mmol/l, 5.6 mmol/l, 16.7 mmol/l or 25 mmol/l), palmitate or oleate (both BSA-conjugated; 150 μmol/l and 500 μmol/l). RNA was isolated 24 h later. Glucose uptake was measured using the Glucose Uptake-Glo assay (Promega, Madison, WI, USA) as per the manufacturer’s instructions.

### Quantitative real-time PCR

RNA was reverse-transcribed using the RevertAid First Strand cDNA Synthesis Kit (Thermo Scientific, Waltham, MA, USA). Quantitative real-time PCR (qPCR) analysis of *Dpp4* (Mm00494538_m1), *Gip* (Mm00433601_m1)*, Insig1* (Mm00463389_m1)*, Pcsk1* (Mm00479023_m1)*, Gcg* (Mm01269055_m1)*, Pyy* (Mm00520716_g1), *Scd1* (Mm00772290_m1), *Sqle* (Mm01198417) and *Srebf2* (Mm01306292_m1), as well as two housekeeping genes (*Hprt1* [Mm03024075_m1] and *Tbp* [Mm01277042_m1]) was performed using the TaqMan Expression PCR Master Mix (Life Technologies) and the ABI Prism 7900 HT system (Applied Biosystems, Foster City, CA, USA). Data analysis was carried out using the 2^−ΔΔct^ method.

### Secretory assays

GLUTag cells were seeded at a density of 250,000 cells per well and gene KD was performed as described above, with secretory assays being conducted 72 h post seeding. For protein inhibition, cells were treated for 24 h prior to the assay being performed. Secretory assays in response to glucose and the phosphodiesterase inhibitor 3-isobutyl-1-methylxanthine (IBMX) were performed as described previously [19]. For secretory assays in response to KCl, cells were washed with PBS and treated with KRB supplemented with 0.1 mmol/l diprotin A (Sigma Aldrich) and 50 mmol/l KCl for 15 min. Following this, supernatant was collected and centrifuged at 500 *g* for 5 min. Cells were lysed using cOmplete Lysis-M (Roche) and total protein concentration was determined using Bio-Rad Protein Assay dye reagent (Bio-Rad Laboratories, Hercules, CA, USA). Supernatant was stored at −20ºC until GLP-1 levels were measured. Concentrations of GLP-1 were determined using the Multi Species GLP-1 Total ELISA (EZGLP1T-36K; Sigma Aldrich), as per the manufacturer’s instructions.

### Imaging of GLUTag cells

For imaging experiments, GLUTag cells were plated on poly-l-lysine-coated (1μg/ml) Lab-Tek eight-well chambered cover glass dishes (#155411, Thermo Scientific) at a density of 60,000 cells/well. Measurements of [Ca^2+^]_c_ were performed as described previously [20] using Fluo4AM (F14201, Thermo Scientific).

For plasma membrane potential recordings, the FLIPR Membrane Potential Explorer Kit Red (including PMPi dye) was used (R8126, Molecular Devices, San Jose, CA, USA). Measurements of plasma membrane potential were performed as described previously [21].

### Cholesterol efflux

GLUTag cells were plated at a density of 150,000 cells per well in 24-well plates and cholesterol efflux was measured as described previously [22].

### Quantification of oxidative phosphorylation

Oxygen consumption rates were measured using the Seahorse XFe24 Extracellular Flux Analyzer (Agilent Technologies, Santa Clara, CA, USA). GLUTag cells were seeded at a density of 20,000 cells per well on poly-l-lysine-coated Seahorse XF cell culture plates. Cells were pre-incubated in a CO_2_-free incubator for 2 h at 37 C in assay buffer (114 mmol/l NaCl, 4.7 mmol/l KCl, 1.2 mmol/l KH_2_PO_4_, 1.16 mmol/l MgSO_4_, 20 mmol/l HEPES and 2.5 mmol/l CaCl_2_, pH 7.2). The respiratory rate was subsequently measured before and after injection of 16.7 mmol/l glucose. Thereafter, a further three components (1 µmol/l oligomycin, 1 µmol/l FCCP and 1 µmol/l rotenone) were injected sequentially. In each instance, as with the initial injection of 16.7 mmol/l glucose, the stabilised cellular respiratory rate was measured to determine changes in mitochondrial respiratory response. With the exception of injection timings, experiments were performed as described previously [23]. Primary data analysis was performed using the Seahorse Analytics webtool (https://seahorseanalytics.agilent.com/; version 1.0.0-570).

### In vivo models

Twelve-week-old female C57Bl/6j mice were sourced from Janvier Labs (Le Genest-Saint-Isle, France) and were housed in a climate-controlled room (23±1°C) with a 12 h/12 h light/dark cycle and fed a normal chow diet. i*Scd1*^−/−^ mice were generated by crossing C57Bl/6j *Scd1*flox/flox mice (kindly donated by J. Ntambi, University of Wisconsin-Madison, Madison, WI, USA to AM) with C57Bl6/j villin-CRE mice. The generation and characterisation of the i*Scd1*^−/−^ mice have been described previously [11]. Two- to three-month-old male i*Scd1*^−/−^ mice and i*Scd1*^+/+^ littermates were kept at 21±2°C with a 12 h/12h light/dark cycle and had free access to food and water. Mice were euthanised using cervical dislocation and the jejunum was collected for both qPCR and immunohistochemistry. All studies were approved by the Regional Ethical Review Boards at Lund University and at the University of Bari ‘Aldo Moro’.

### OGTTs

Prior to performing OGTTs, mice were fasted for 4 h (starting at 0700). Mice were anaesthetised by intraperitoneal injection of Hypnorm/Dormicum (10 μg/l body weight; fentanyl 0.315 mg/ml, fluanison 10 mg/ml and midazolam 5 mg/ml) or Lobotor (100 mg/kg body weight; 100 mg/ml ketamine hydrochloride) and xylazine (5 mg/kg body weight). Anaesthesia was chosen based on the ethical permission granted to the laboratory where the OGTT was performed. A total of 500 μg/kg SCD1 inhibitor (Ab142089, Abcam) or 0.9% NaCl (wt/vol.) was administered orally 1 h pre OGTT to lean C57Bl/6j female mice (*n*=10), which were housed in groups of five mice per cage and weighed 23–31 g. Animals were chosen randomly to receive each treatment. The concentration of SCD1 inhibitor was chosen as it was similar to that shown to reduce tumour growth in a mouse xenograft model [24]. OGTTs in i*Scd1*^−/−^ and i*Scd1*^*+*/+^ mice were performed in male mice (*n*=10 per group), which were housed in groups of three to five mice per cage and weighed 24–33 g. Basal blood samples were collected by retro-orbital puncture (40 μl), after which 3 g/kg glucose was administered orally. Animals were blinded to the experimenter. Samples were collected in EDTA-coated tubes, which were further coated with 0.1 mmol/l Ile-Pro-Ile (Sigma Aldrich). Blood samples were collected by retro-orbital puncture at 5, 10, 15, 30, 60 and 90 min post gavage and centrifuged at 4700 *g* at 4ºC for 2 min. Plasma was stored at −80ºC until analysis. Glucose levels were analysed using an AccuChek Aviva glucose meter (Roche) and total GLP-1 was analysed using the Multi Species GLP-1 Total ELISA.

### Statistical analyses

All statistical analyses were performed in Graphpad Prism 9.0 (GraphPad Software, La Jolla, CA, USA). For paired analyses, two-tailed *t* tests were performed, while for grouped analyses, ordinary one-way ANOVA with Dunnett’s multiple comparisons test with a single pooled variance was performed. Data are presented as mean + SEM.

## Results

### RYGB-induced changes in jejunal gene expression

To perform an unbiased assessment of the transcriptomic influence of RYGB on the jejunal mucosa, biopsies (from the same site) taken from 13 participants [5] (Table 1) perisurgically and 1 year post RYGB were analysed using RNA-seq. Differential gene expression analysis identified 614 differentially expressed genes of 14,271 genes examined (electronic supplementary material [ESM] Table 1). GO enrichment analysis of the 614 differentially expressed genes identified 103 upregulated pathways (ESM Table 2, ESM Fig. 1) and eight downregulated pathways (ESM Table 3, ESM Fig. 1). The pathway most highly enriched following RYGB was cholesterol import, which was markedly upregulated. The most significantly affected gene following RYGB was insulin-induced gene 1 (*INSIG1*, fold change [FC] 2.7, *p*_adj_=4.3×10^−22^)*. SCD* (FC 2.5, *p*_adj_ 3.4×10^−10^), which encodes an enzyme responsible for the synthesis of monounsaturated fatty acids [25], and *SQLE* (FC 2.1, *p*_adj_=6.8×10^−10^), which encodes squalene epoxidase (SQLE), one of the key enzymes in cholesterol biosynthesis [26], were also among the most significantly upregulated genes. INSIG1 protein binds to a sterol-sensing domain of sterol regulatory element-binding (SREB) proteins [27] and, in line with this, *SREBF2*, the gene encoding sterol regulatory element-binding transcription factor 2 (SREBF2) was also upregulated (FC 0.6, *p*_adj_=0.03).

### Expression in human L cells

**Fig. 1:**
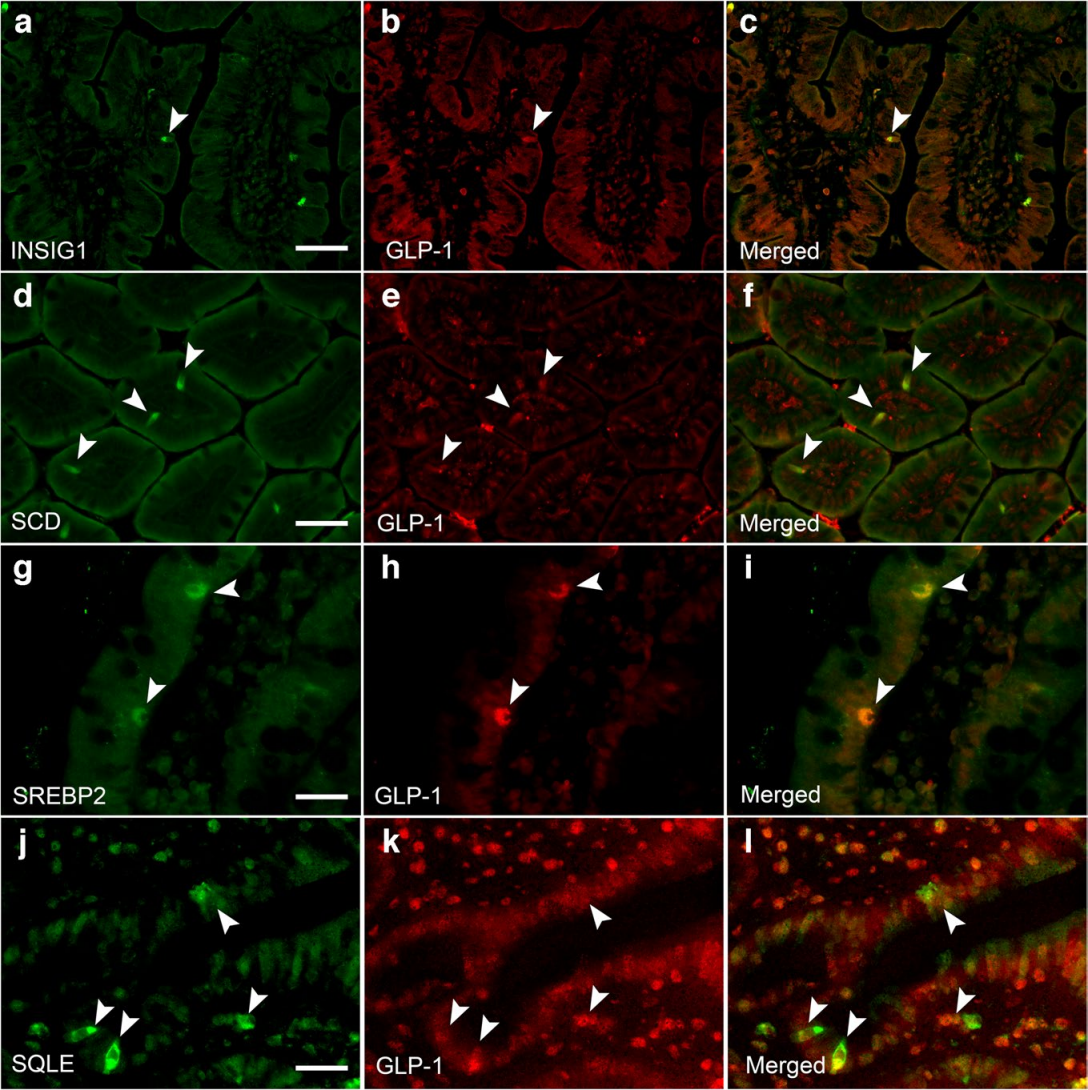
Immunohistochemistry of human jejunum identifying the presence of INSIG1 (**a**), SCD (**d**) SREBP2 (**g**) and SQLE (**j**) in GLP-1-producing cells (**b**, **e**, **h**, **k**). Merge of both panels shown in (**c**), (**f**), (**i**) and (**l**). Immunohistochemistry was repeated in three separate samples

Although it is well established that RYGB causes increased GLP-1 secretion [28], the underlying mechanisms are not yet fully understood. We hypothesised that genes highly affected by RYGB could be novel regulators of GLP-1 secretion and production. *INSIG1*, *SCD*, *SQLE* and *SREBF2* were chosen as candidate genes based on their differential expression and key roles in cholesterol pathways. Immunohistochemical analysis confirmed expression of the corresponding protein products in human jejunal L cells (Fig. 1), while gene expression of both *GCG* and *SCD* was further confirmed in the same cell type using a published single-cell RNA sequencing (scRNA-seq) dataset of human intestinal cells [29] via cellxgene (version 1.1.1) [30] (mean log *SCD* transcripts per million [TPM] was 4.3 in *GCG* and *SCD* co-expressing cells; ESM Fig. 2).

### Effects of target genes on GLP-1 production in GLUTag cells

**Fig. 2:**
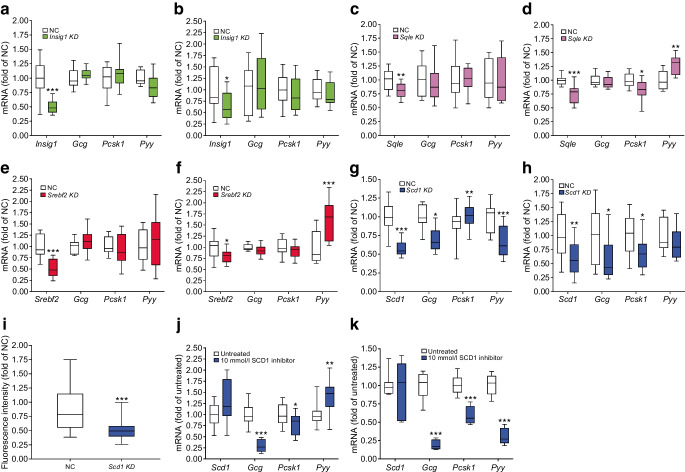
Regulation of *Gcg*, *Pcsk1* and *Pyy* mRNA in GLUTag cells and STC-1 cells. KD of *Insig1* (**a**), *Sqle* (**c**), *Srebf2* (**e**) and *Scd1* (**g**) in GLUTag cells. *Scd1* KD reduced *Gcg* and *Pyy* expression while increasing *Pcsk1* expression (**g**). KD of *Insig1* (**b**), *Sqle* (**d**), *Srebf2* (**f**) and *Scd1* (**h**) was replicated in STC-1 cells. *Scd1* KD reduced *Gcg* and *Pcsk1* expression (**h**). (**i**) Quantification of immunoreactive protein after *Scd1* KD in GLUTag cells. SCD1 protein was inhibited in GLUTag (**j**) and STC-1 cells (**k**). SCD1 inhibition decreased *Gcg* and *Pcsk1* expression and increased *Pyy* expression in GLUTag cells, while the expression of all three genes was reduced in STC-1 cells. Data are presented as box and whisker plots showing the minimum and maximum values (bottom and top error bars), the first (bottom of the box) and third (top of the box) quartiles and the median (middle of the box). The graphs show fold change relative to NC or untreated cells for each gene. All groups were normalised to NC or untreated cells. Experiments were performed in duplicate in six separate passages of cells. *p<0.05, **p<0.01 and ***p<0.001 compared with negative control or untreated cells. NC, negative control

Having established the expression of *INSIG1*, *SCD*, *SQLE* and *SREBF2* in human L cells, we next investigated the potential influence of these genes on *Gcg* mRNA using siRNA-mediated KD in GLUTag cells. This cell line expresses *Gcg*, the gene for proglucagon; *Pyy,* which encodes an appetite-suppressing hormone [31]; and *Pcsk1*, encoding proprotein convertase 1, which cleaves GLP-1 from the proglucagon precursor protein [32, 33]. Expression of the selected genes in GLUTag cells was verified using RNA-seq (mean TPM for *Insig1*, *Scd1*, *Sqle* and *Srebf2* were 3416, 2157, 2549 and 2187, respectively; data not shown). *Scd1* KD reduced *Gcg* and *Pyy* expression (Fig. 2g). KD of *Insig1* (49.4% reduction; *p*<0.001; Fig. 2a), *Sqle* (19.8% reduction; *p*<0.01; Fig. 2c) or *Srebf2* (47.4% reduction; *p*<0.001; Fig. 2e) had no effect on any of the genes investigated. Immunocytochemistry confirmed that *Scd1* KD reduced SCD1 protein levels in GLUTag cells (Fig. 2i). We repeated the siRNA experiments in a second hormone-producing intestinal cell line; STC-1 cells [34, 35]. *Scd1* KD reduced *Gcg* and *Pcsk1* expression (Fig. 2h). KD of *Insig1* (35.4% reduction; *p*<0.05; Fig. 2b)*, Sqle* (23.5% reduction; *p*<0.001; Fig. 2d) or *Srebf2* KD (19.5% reduction; *p*<0.05; Fig. 2f) had no effect on *Gcg* expression.

### Effects of SCD1 inhibition on GLP-1 production from GLUTag cells

Next, we used a commercially available SCD1 inhibitor and assessed its effect on *Gcg* expression and total GLP-1 secretion in GLUTag cells. Addition of 10 mmol/l SCD1 inhibitor for 24 h to GLUTag cells resulted in a reduction in *Gcg* (*p*<0.001) and *Pcsk1* (*p*<0.05) mRNA expression (Fig. 2j). Conversely, *Pyy* mRNA expression was increased 1.4-fold (*p*<0.01). In STC-1 cells, addition of 10 mmol/l SCD1 inhibitor resulted in reductions in *Gcg*, *Pcsk1* and *Pyy* mRNA expression (Fig. 2k; *p*<0.001).

### Effect of ***Scd1*** knockdown and SCD1 inhibition on GLP-1 secretion in GLUTag cells

**Fig. 3:**
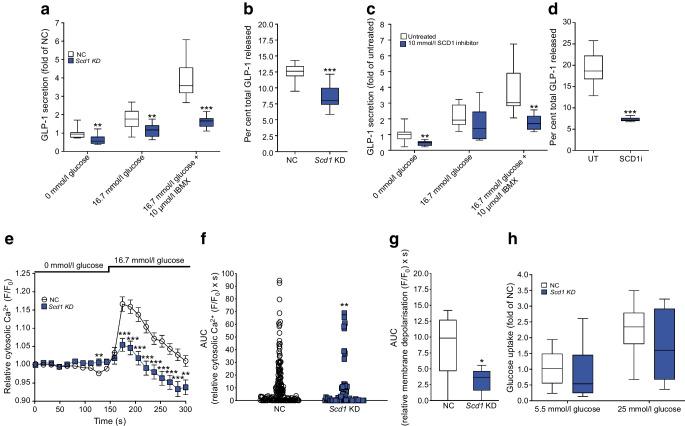
Effect of *Scd1* KD and SCD1 inhibition on GLP-1 secretion and exocytosis from GLUTag cells. (**a**) In GLUTag cells treated with 0 mmol/l, 16.7 mmol/l glucose and 16.7 mmol/l glucose plus 10 μmol/l IBMX, *Scd1* KD resulted in decreased GLP-1 secretion compared with the negative control. (**b**) *Scd1* KD also resulted in a reduction in GLP-1 secretion in the presence of 50 mmol/l KCl. SCD1 inhibition reduced GLP-1 secretion from GLUTag cells in response to 0 mmol/l glucose and 16.7 mmol/l glucose plus 10 μmol/l IBMX (**c**) as well as 50 mmol/l KCl (**d**). *Scd1* KD reduced intracellular calcium signalling as measured by Fluo4AM (**e**, **f**), as well as membrane depolarisation as measured using PMPi (**g**). (**h**) *Scd1* KD had no effect on glucose uptake. Data are presented as box and whisker plots showing the minimum and maximum values (bottom and top error bars), the first (bottom of the box) and third (top of the box) quartiles and the median (middle of the box). (**a**, **c**, **h**) Fold change relative to NC at 0 mmol/l (**a**), untreated cells at 0 mmol/l glucose (**b**) and NC at 5.5 mmol/l (**c**). All groups were normalised to NC or untreated cells. GLP-1 secretory assays were performed in duplicate in six separate passages of cells. For intracellular calcium signalling and membrane depolarisation imaging, five passages of cells were used for NC and six for *Scd1* KD and each replicate was measured repeatedly over the time course of the experiment. *p<0.05, **p<0.01 and ***p<0.001 compared with negative control or untreated cells at the same glucose treatment. NC, negative control; SCD1i, 10 mmol/l SCD1 inhibitor; UT, untreated

As *Scd1* KD and SCD1 inhibition affected *Gcg* expression in GLUTag and STC-1 cells, this gene was chosen for further examination. First, we assessed the effects of *Scd1* KD on total GLP-1 secretion from GLUTag cells in static incubations. These cells have been shown to secrete GLP-1 [36], thus providing a suitable model system. *Scd1* KD caused a 35% reduction (*p*<0.01) in GLP-1 secretion at 0 mmol/l and 16.7 mmol/l glucose respectively (Fig. 3a), a 58% reduction (*p*<0.01) in cells treated with 16.7 mmol/l glucose and 10 μmol/l IBMX (stimulator of cAMP [37]), and a 31% reduction in cells treated with 50 mmol/l KCl, which induces membrane depolarisation [38] in the absence of glucose (Fig. 3b). Furthermore, SCD1 inhibition resulted in a 55% and 54% reduction in GLP-1 secretion in response to 0 mmol/l and 16.7 mmol/l glucose plus 10 μmol/l IBMX, respectively (Fig. 3c; *p*<0.01). Further mirroring the siRNA data, SCD1 inhibition caused a 62% reduction in GLP-1 secretion triggered by 50 mmol/l KCl in the absence of glucose (Fig. 3d; *p*<0.001).

To understand whether the effects of *Scd1* KD on total GLP-1 secretion were due to reduced exocytosis, we performed imaging of cells after *Scd1* KD using Fluo4AM (intracellular Ca^2+^) and PMPi (membrane depolarisation) dyes. Intracellular Ca^2+^ imaging (Fig. 3e) showed that *Scd1* KD resulted in a 26% reduction in Ca^2+^ concentration following the addition of 16.7 mmol/l glucose (Fig. 3f; *p*<0.01) based on the AUC. In agreement with this, membrane depolarisation imaging showed that *Scd1* KD resulted in a 65% reduction in depolarisation (Fig. 3g; *p*<0.05) following addition of 16.7 mmol/l glucose. Furthermore, we assessed the effect of *Scd1* KD on glucose uptake in GLUTag cells but found no effect during normo- or hyperglycaemic conditions (Fig. 3h). Therefore, it is likely that the observed effects on GLP-1 secretion in response to *Scd1* KD are at least in part related to direct effects on intracellular events that are affected by calcium signalling and membrane depolarisation. However, altered GLP-1 synthesis cannot be completely ruled out.

### RNA sequencing of GLUTag cells treated with SCD1 inhibitor

To gain further insight into the cellular response mediated by SCD1 inhibition, we performed RNA-seq on GLUTag cells treated with 10 mmol/l SCD1 inhibitor for 24 h. SCD1 inhibition caused upregulation of 2051 and downregulation of 2225 genes of 20,766 genes examined (ESM Table 4). In agreement with our PCR data, *Gcg* and *Pcsk1* were among the downregulated genes. GO term analysis revealed no significant upregulated GO terms. However, GO term analysis of downregulated genes alone identified processes related to glycolysis and ATP generation as being highly enriched (ESM Table 5).

### Regulation of ***Scd1*** by lipids and glucose

**Fig. 4:**
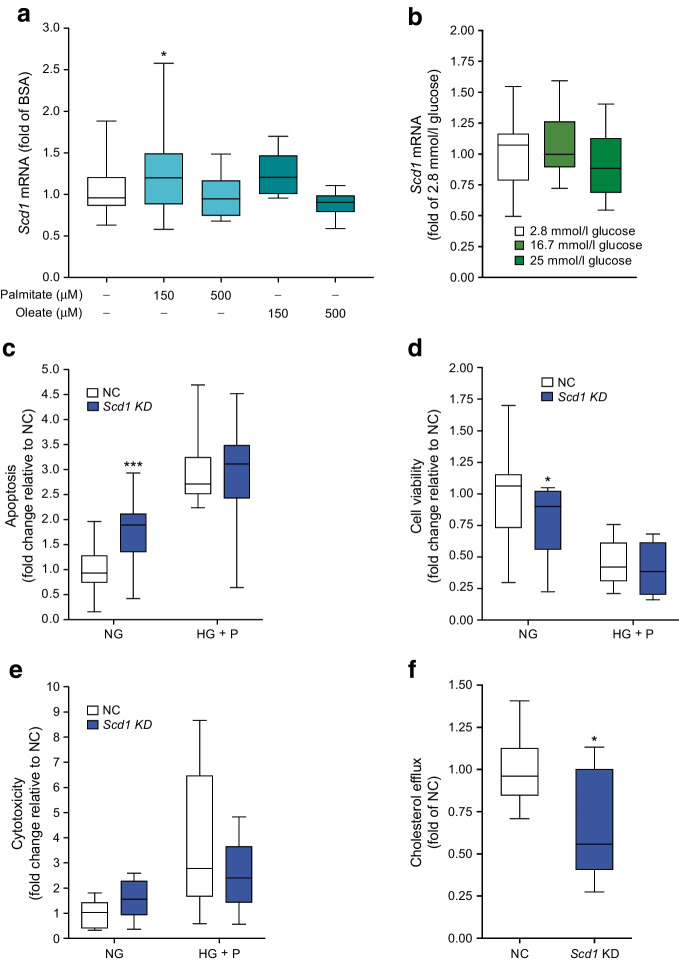
Regulation of *Scd1* mRNA and the effect of *Scd1* on cholesterol efflux in GLUTag cells. (**a**) Treatment of GLUTag cells for 24 h (5.5. mmol/l glucose) with 150 μmol/l palmitate increased *Scd1* expression. The white bars denote the vehicle control (BSA). (**b**) Glucose had no effect on *Scd1* expression at any of the concentrations tested. Apoptosis was increased under basal conditions in response to *Scd1* KD (**c**) while cell viability was reduced (**d**), with no effects on cytotoxicity being observed (**e**). (**f**) *Scd1* KD reduced cholesterol efflux in GLUTag cells. Data are presented as box and whisker plots showing the minimum and maximum values (bottom and top error bars), the first (bottom of the box) and third (top of the box) quartiles and the median (middle of the box). (**c**, **d**, **e**) Fold change relative to NC under the condition NG. All groups were normalised to NC. Experiments were performed in duplicate in six separate passages of cells. *p<0.05 and ***p<0.001 compared with negative control or untreated cells at the same glucose treatment. HG + P, high glucose (25 mmol/l) with high palmitate (500 μmol/l); NC, negative control; NG, no glucose

Intestinal L cells sense a number of different stimuli including glucose and fatty acids [39]. To test whether *Scd1* expression is affected by fatty acids and glucose, GLUTag cells were treated with oleate (150 μmol/l, 500 μmol/l), palmitate (150 μmol/l, 500 μmol/l; Fig. 4a), which we have reported previously [19], or glucose (2.8, 16.7 or 25 mmol/l for 24 h; Fig. 4b). *Scd1* expression was unaffected by glucose and oleate but increased 1.3-fold in response to 150 µmol/l palmitate (Fig. 4a; *p*<0.05).

### Effects of ***Scd1*** knockdown on apoptosis, cytotoxicity, cell viability and cholesterol efflux

In order to understand whether the observed effects of *Scd1* KD on GLP-1 secretion could be related to alterations in cell viability, cytotoxicity or apoptosis, an ApoTox assay was carried out in GLUTag cells cultured under normal (5.5 mmol/l glucose) or glucolipotoxic (25 mmol/l glucose with 500 μmol/l palmitate) conditions for 24 h. While *Scd1* KD had no effect on cell viability, apoptosis or cytotoxicity under toxic culture conditions, under normal culture conditions (5.5 mmol/l glucose), *Scd1* KD resulted in a 1.79-fold increase in apoptosis in (*p*<0.001; Fig. 4c) and a 22% decrease in cell viability (*p*<0.05; Fig. 4d). No effects were observed on cytotoxicity (Fig. 4e). Further to this, *Scd1* KD reduced cholesterol efflux from GLUTag cells by 32% (Fig. 4f).

### Effect of ***Scd1*** knockdown on respiration

**Fig. 5:**
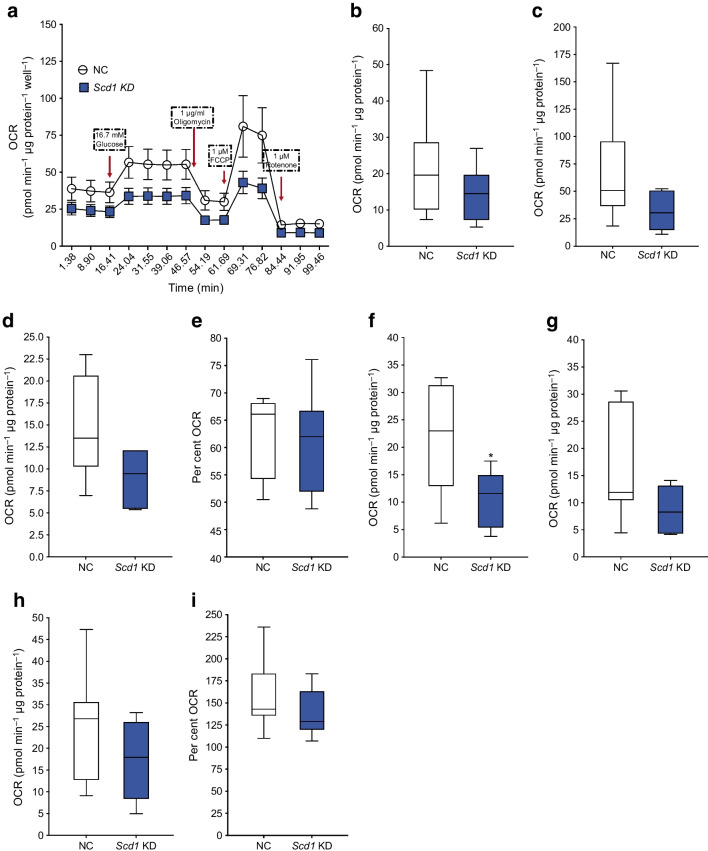
The effect of *Scd1* KD on respiration in GLUTag cells. Respiration in response to *Scd1* KD in GLUTag cells was assessed using a Seahorse XFe24 Extracellular Flux Analyzer. (**a**) Trace of the whole experiment; (**b**) basal respiration; (**c**) maximal respiration; (**d**) non-mitochondrial oxygen consumption; (**e**) coupling efficiency; (**f**) acute response; (**g**) proton leak; (**h**) ATP production and (**i**) spare respiratory capacity. Data are presented as box and whisker plots showing the minimum and maximum values (bottom and top error bars), the first (bottom of the box) and third (top of the box) quartiles and the median (middle of the box). Experiments were performed with five technical replicates per condition (NC and *Scd1* KD) in six separate passages of cells. Each replicate was measured repeatedly over the time course of the experiment. *p<0.05 compared with negative control. FCCP, carbonyl cyanide-4 (trifluoromethoxy) phenylhydrazone; NC, negative control; OCR, oxygen consumption rate

The effect of *Scd1* KD on cell respiration was assessed using a Seahorse XF cell mito stress test. *Scd1* KD resulted in a non-significant reduced respiratory capacity over the course of the test (*p*=0.10–0.76; Fig. 5a). Several calculated variables, such as maximal respiration (*p*=0.13), non-mitochondrial oxygen consumption (*p*=0.05) and proton leak (*p*=0.06), also showed non-significant reductions (Fig. 5). Most notably, the acute response was reduced by 48% in response to *Scd1* KD (*p*<0.05; Fig. 5f), suggesting a reduction in the respiratory capacity of the mitochondria in GLUTag cells in response to glucose. However, there was a downward shift in basal respiration as a result of *Scd1* KD. When the baseline was corrected and fold change was measured, it became apparent that the effects of *Scd1* KD on basal respiration were the driving force of the observed results (data not shown).

### Effects of SCD1 inhibition in vivo

**Fig. 6:**
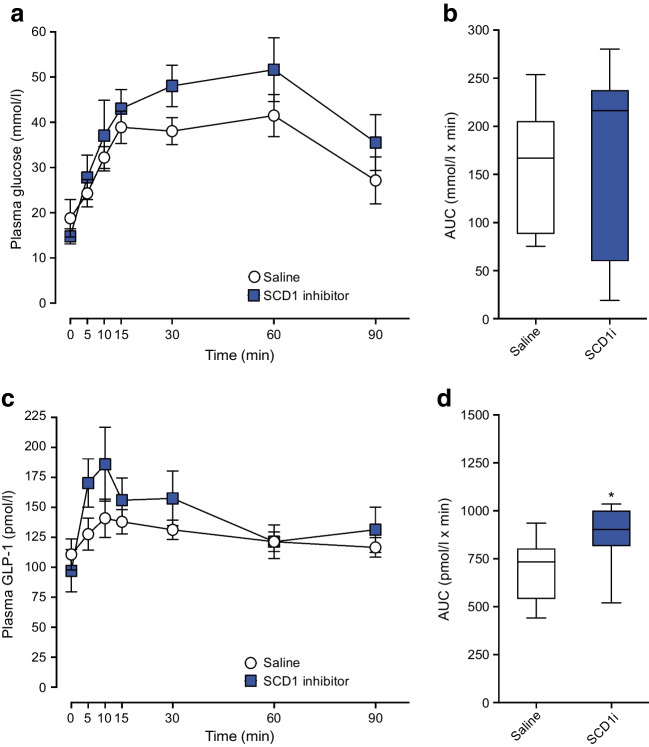
Effects of SCD1 inhibition in vivo*.* Mice were gavaged with either saline or 500 μg/kg SCD1 inhibitor 1 h prior to an OGTT. SDC1 inhibition had no effect on plasma glucose (**a**, **b**), or plasma GLP-1 at individual time points (**c**), but a moderate increase in GLP-1 AUC (**d**) was observed. Data are presented as box and whisker plots showing the minimum and maximum values (bottom and top error bars), the first (bottom of the box) and third (top of the box) quartiles and the median (middle of the box). The OGTT was performed in ten mice per treatment group. *p<0.05 compared with untreated mice. SCD1i, 500 μg/kg SCD1 inhibitor

We next assessed whether SCD1 affects total GLP-1 secretion in vivo. To this end, mice were gavaged with 500 µg/kg SCD1 inhibitor or vehicle 1 h prior to an OGTT. SCD1 inhibition had no effect on glucose levels (Fig. 6a,b) or GLP-1 levels (Fig. 6c) at individual time points; however, an overall moderate increase (1.2-fold) in the AUC for GLP-1 was observed (Fig. 6d).

### Effects of ***Scd1*** knockout in vivo

**Fig. 7:**
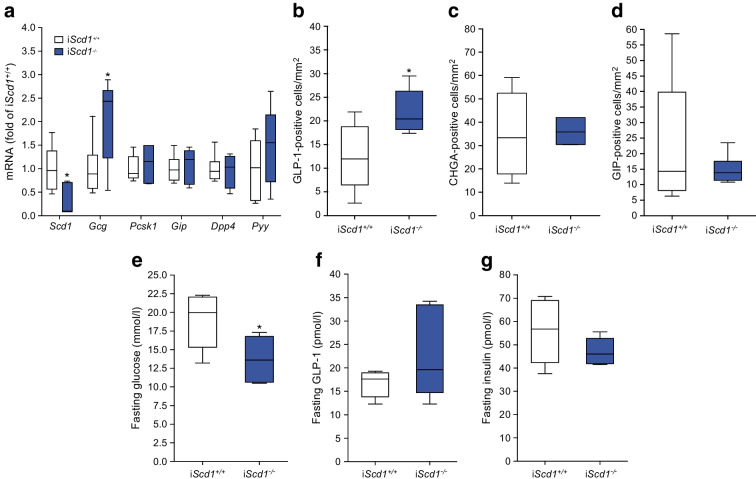
Effects of intestinal-specific knockout of *Scd1* (i*Scd1*^−/−^) in vivo. Compared with i*Scd1*^+/+^ mice, i*Scd1*^−/−^ mice demonstrated increased jejunal *Gcg* gene expression (**a**) and increased jejunal GLP-1-positive cell number (**b**), with no effects observed on chromogranin A (CHGA)-positive cell number (**c**) or GIP-positive cell number (**d**). i*Scd1*^−/−^ mice had lower fasting plasma glucose levels (**e**), while fasting GLP-1 (**f**) and fasting insulin (**g**) levels were unaffected. Data are presented as box and whisker plots showing the minimum and maximum values (bottom and top error bars), the first (bottom of the box) and third (top of the box) quartiles and the median (middle of the box). (**a**) Fold change relative to i*Scd1*^+/+^ for each gene. All groups were normalised to i*Scd1*^+/+^. Experiments were performed in five control mice and six i*Scd1*^−/−^ mice. *p<0.05 compared with i*Scd1*^+/+^ mice

To further address the role of SCD1 in vivo, we used intestinal-specific *Scd1* knockout (i*Scd1*^−/−^) mice [11]. Total GLP-1 secretion and glucose levels were unaffected in i*Scd1*^−/−^ mice during an OGTT (ESM Fig. 3), but jejunal *Gcg* expression (Fig. 7a) and L cell density (Fig. 7b) were 2- and 1.8-fold higher (*p*<0.05) in i*Scd1*^−/−^ mice compared with i*Scd1*^+/+^ mice. The latter effect seemed specific to L cells, as there was no alteration in chromogranin A (CHGA; pan-endocrine marker)-positive cells (Fig. 7c) or glucose-dependent insulinotropic peptide (GIP)-positive cells (Fig. 7d) in i*Scd1*^−/−^ mice. Furthermore, i*Scd1*^−/−^ mice demonstrated a 28% lower fasting glucose level than i*Scd1*^+/+^ mice (Fig. 7e), but GLP-1 (Fig. 7f) and insulin (Fig. 7g) levels were unaffected.

## Discussion

It is well established that the postprandial GLP-1 response is increased after RYGB [40]; however, the mechanisms behind this have yet to be elucidated. In this study we used an unbiased transcriptomic approach to identify potential regulators of GLP-1 in a segment of the intestine chosen for its accessibility both during surgery and post surgery. RYGB caused major alterations in the jejunal transcriptome, and genes related to cholesterol biosynthesis stood out as being most affected. We tested the possibility that genes highly affected by RYGB could play a role in intestinal L cell function and the regulation of GLP-1 secretion. In a series of experiments, we show that the RYGB-regulated enzyme SCD, a regulator of cholesterol levels [41], acts as a regulator of GLP-1 transcription and secretion.

To the best of our knowledge, global transcriptomic effects of RYGB on the human intestine have been described only twice previously [42, 43]. Neither of these studies included functional follow-up of identified targets. There are several differences between these studies and ours. Some of the effects of RYGB have recently been attributed to the pre-operation very low-energy diet (very low calorie diet [VLCD]) [7] and, as Jorsal et al [42] collected biopsies 3 months pre surgery, the influence of a VLCD could not be excluded. Furthermore, we collected biopsies 1 year apart whereas Jorsal et al [42] collected biopsies 6 months apart. Individuals have a varied, albeit reduced, diet 1 year post RYGB, whereas this is not normally the case at 3 months. Sala et al [43] studied female participants with type 2 diabetes whereas the participants in our study had not been diagnosed with type 2 diabetes. Despite these differences, the data presented here and in both previous studies, as well as in a previous microarray study [44], identified sterol biosynthesis or genes involved in these processes as being greatly affected by RYGB. Further to this, a recent global proteomics study by Wallenius et al found mitochondrial 3-hydroxy-3-methylglutaryl-CoA synthase (mHMGCS) to be robustly affected by RYGB [45]. This study differed from ours as we adopted a transcriptomics rather than proteomics approach and biopsies were collected at different time points. The fact that different approaches still identified genes and proteins involved in cholesterol biosynthesis as being affected demonstrates the robustness of the effects of RYGB on cholesterol biosynthesis.

*SCD* encodes a rate-limiting enzyme involved in the regulation of monounsaturated fatty acid synthesis [46] and is expressed ubiquitously [47]. *SCD* is essential for normal cellular function [46] and has been implicated in a number of inflammatory diseases, including obesity and insulin resistance [48]. In our dataset, *SCD* was among the most significantly differentially expressed genes after RYGB (ranked 5 of 614 genes). Expression of the other human *SCD* isoform, *SCD5* [46], was unaffected by RYGB.

Using GLUTag and STC-1 cells as model systems, we found that both siRNA-mediated KD and pharmacological inhibition of SCD1 reduce GLP-1 production and secretion. The present finding that SCD1 inhibition reduces total GLP-1 secretion under all conditions tested, including in the presence of K^+^, points towards an effect on late events in GLP-1 release. Supporting this notion, *Scd1* KD resulted in reductions in membrane depolarisation and glucose-induced intracellular calcium concentrations. Furthermore, we found that *Scd1* expression in GLUTag cells was unaffected by exposure to glucose but increased by palmitate. This is in line with the role of SCD1 as a key enzyme in the synthesis of monounsaturated fatty acids, with its main substrates being palmitic and stearic acids [46]. Further supporting a role for SCD1 in the stimulus–secretion coupling chain in L cells, SCD1 inhibition was found to reduce the expression of genes related to glycolysis, as well as ATP and NADH production. These are processes that have previously been shown to affect L cell secretion [49, 50]. In agreement, we found that *Scd1* KD reduced the acute mitochondrial respiratory response as measured using the Seahorse assay. However, *Scd1* KD reduced basal respiration and, when this was corrected for, it was found that the effects on basal respiration were the driving force for the observed effects. Thus, our in vitro data suggest that *SCD* is a regulator of GLP-1 secretion through direct effects on L cell metabolism.

Surprisingly, acute oral administration of an SCD1 inhibitor caused a moderate elevation of total plasma GLP-1 in vivo in mice. Furthermore, intestinal-specific i*Scd1*^−/−^ mice had unaffected fasting and postprandial GLP-1 levels during an OGTT. Thus, although SCD1 inhibition clearly exerts direct effects in L cells, these effects are not seen at the whole-body level, potentially related to the ubiquitous expression of SCD1. Deletion of *Scd1* reduces the synthesis of oleic and palmitoleic acids and thus prevents the accumulation of lipids in cells [51]. A recent study has shown that glucolipotoxic conditions can reduce GLP-1 secretion [52]; therefore, increased levels of intracellular lipids may be detrimental to GLP-1 secretion. In support of this, whole-body knockout of *Scd1* results in the accumulation of lipids in the liver, causing hepatic steatosis and hyperlipidaemia [41]. Mice express four different SCD isoforms (*Scd1*, *Scd2*, *Scd3* and *Scd4*) in a variety of tissues [53] and it is not known how these isoforms compensate for the loss or inhibition of *Scd1*. Nevertheless, despite having unaffected circulating total GLP-1 levels, i*Scd1*^−/−^ mice displayed robustly elevated *Gcg* mRNA levels, as well as increased L cell density. These data support the presence of increased GLP-1 production in i*Scd1*^−/−^ mice. Given our observation of perturbed GLP-1 production and secretion after inhibition of *Scd1* in cell lines, it is not inconceivable that the observed increased production in i*Scd1*^−/−^ mice is part of a mechanism that compensates for insufficient GLP-1 release by individual L cells.

In summary, using an unbiased approach we have identified novel L cell constituents and multiple genes affected by RYGB in the jejunum. Our functional validation suggests that SCD is a novel regulator of GLP-1 production and secretion. Our findings may promote future pharmacological targeting of L cell regulators as a novel therapeutic approach in type 2 diabetes.

### Supplementary Information

Below is the link to the electronic supplementary material.

Supplementary file1 (PDF 1.82 MB)

Supplementary file2 (XLSX 1473 KB)

## Data Availability

All data are available from the corresponding author on request.

## Abbreviations

CHGA: Chromogranin A
FC: Fold change
GIP: Glucose-dependent insulinotropic peptide
GLP-1: Glucagon-like peptide-1
GO: Gene Ontology
IBMX: 3-isobutyl-1-methylxanthine
KD: Knockdown
qPCR: Quantitative real-time PCR
RYGB: Roux-en-Y gastric bypass surgery
SCD: Stearoyl-CoA desaturase
scRNA-seq: Single-cell RNA sequencing
SQLE: Squalene epoxidase
SREBF2: Sterol regulatory element-binding transcription factor 2
TGR5: Takeda G-protein receptor 5
TPM: Transcripts per million
VLCD: Very low calorie diet
