# Supplementary material for: RNA sequencing unravels novel L cell constituents and mechanisms of GLP-1 secretion in human gastric bypass-operated intestine
Source: Diabetologia. 2023 Nov 30;67(2):356–70. doi: 10.1007/s00125-023-06046-8 (PMC10789678; doi:10.1007/s00125-023-06046-8)
Supplement: Supplementary file 1 — Supplementary file1 (PDF 1.82 MB) [file 125_2023_6046_MOESM1_ESM.pdf]

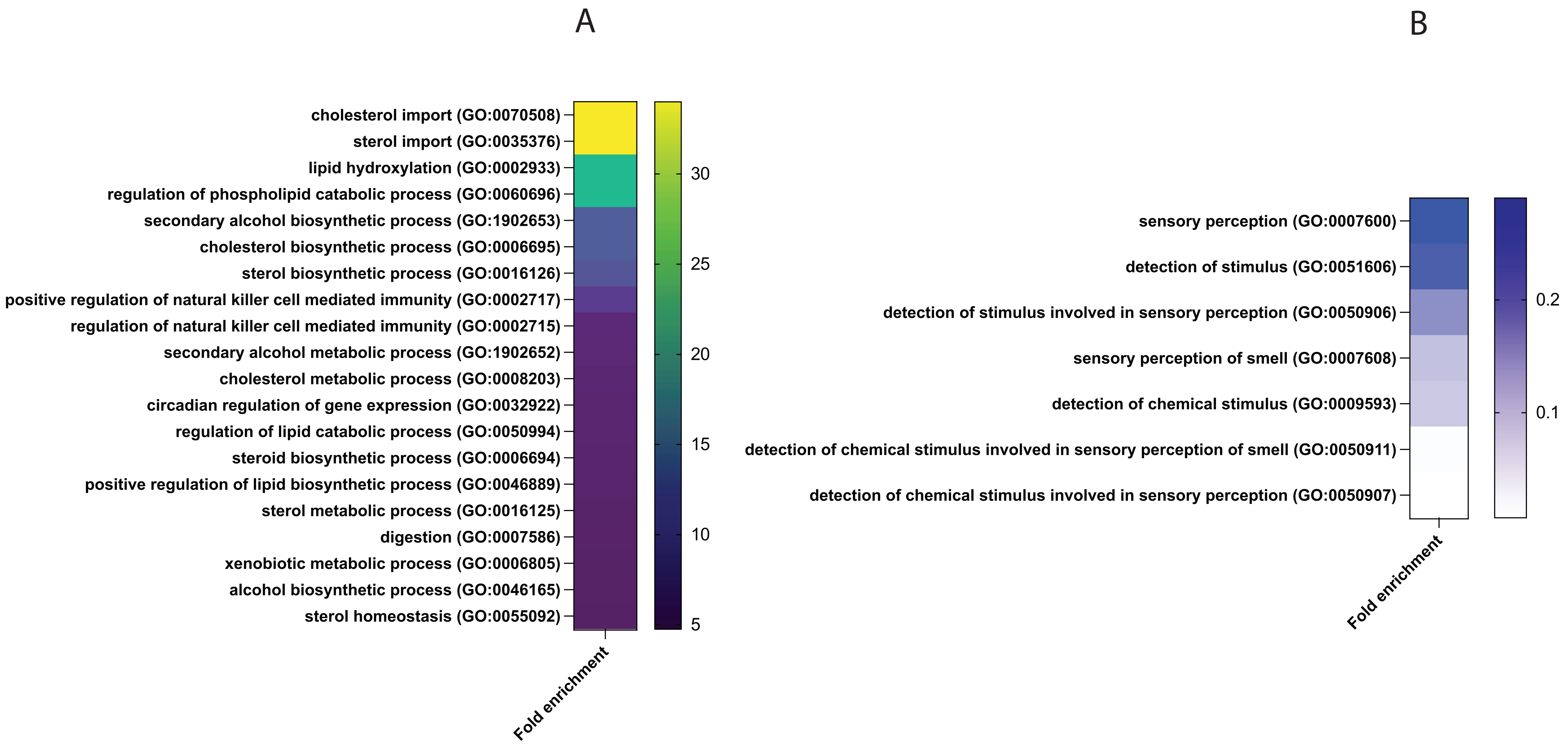

ESM Figure 1. Heat maps showing GO terms up- (A) and down-regulated (B) based on fold enrichment. The 20 most enriched processes are shown in A.

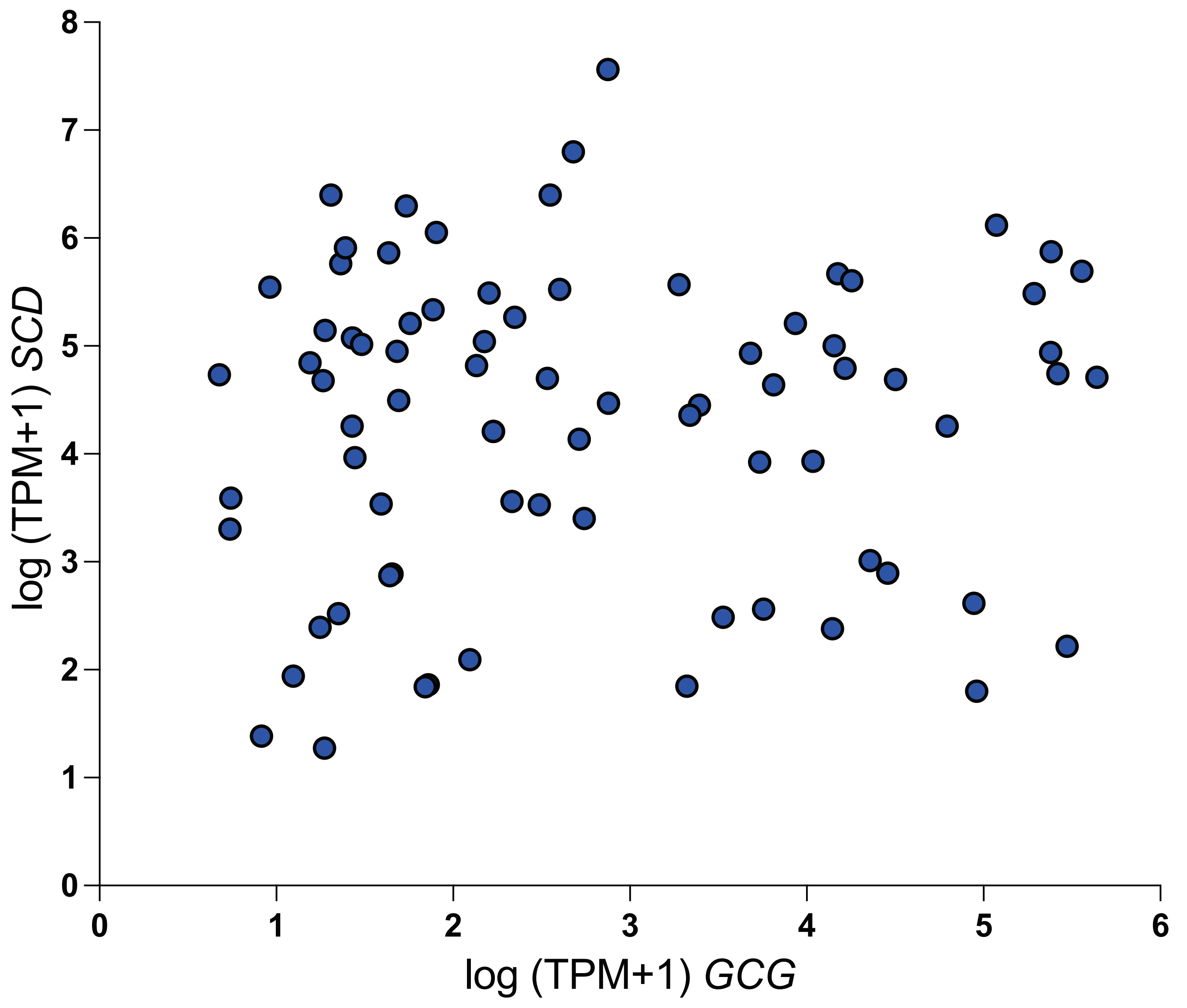

ESM Figure 2. Single-cell RNA sequencing data showing SCD and GCG expression in human intestinal cells.

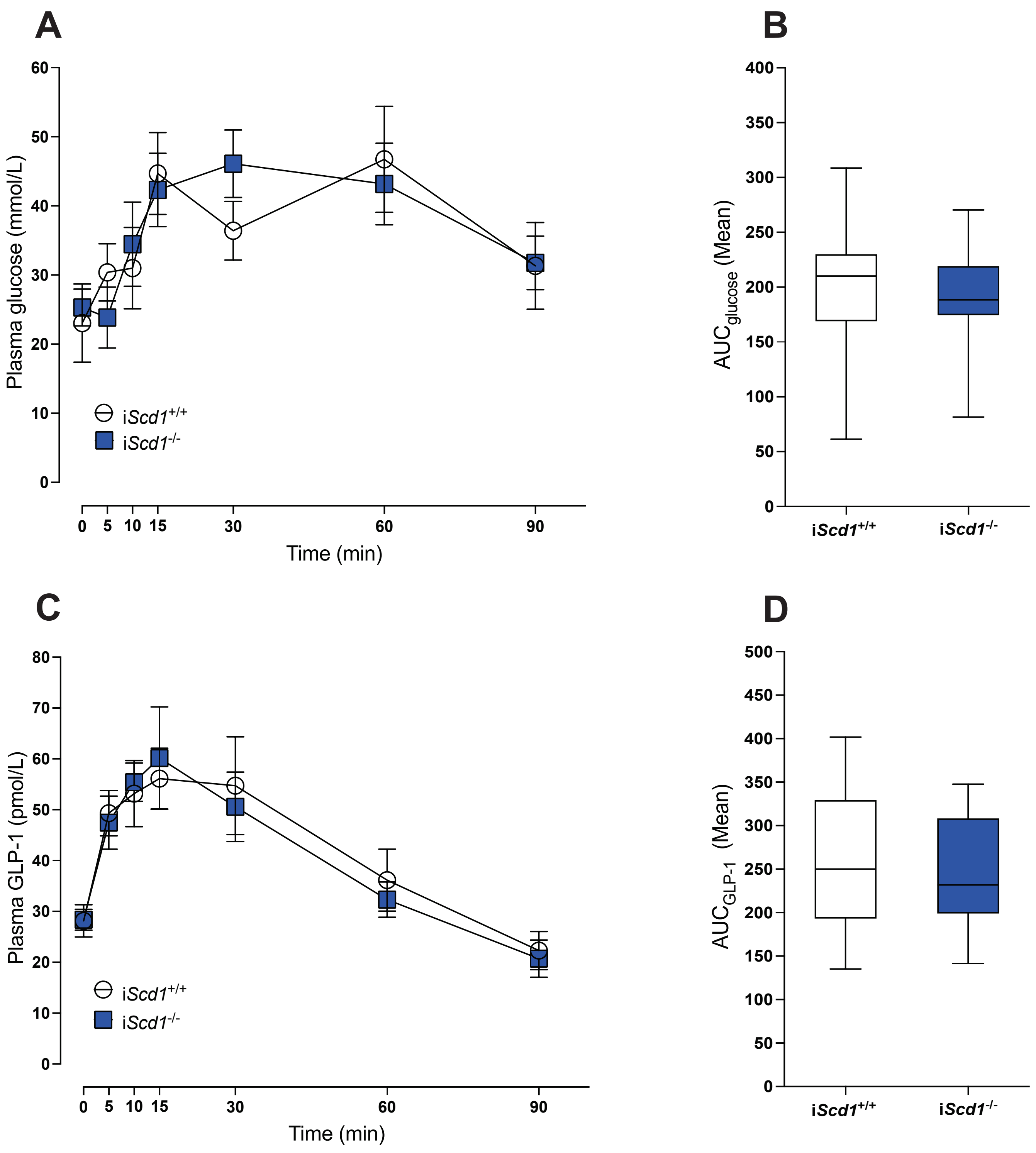

ESM Figure 3. Plasma levels of glucose (A and B) and GLP-1 (C and D) were similar in i*Scd1*$^{-/-}$ mice and wild-type i*Scd1*$^{+/+}$ littermates during an OGTT.
